# Recent Researches on Poliomyelitis

**Published:** 1913-03-01

**Authors:** 


### RECENT RESEARCHES ON POLIOMYELITIS.

Poliomyelitis has for many years been looked on as an occasional degeneration of the spinal cord in children, causing paralysis and deformities such as the celebrated lame foot of Sir Walter Scott. Of late our point of view has entirely changed since it has been shown to be an ordinary infectious general fever, not only producing the classical paralyses, but affecting the liver, the lymphoid, and nervous tissues both in adults and children. The actual mortality is given by Wickman as over 16 per cent., or about that of typhoid or pneumonia, but in addition to this most of the cases which do recover are crippled for life. Even if we think that Wickman's figures are excessive, it is still a disease with a very serious outlook. Besides this there are grounds for thinking it is becoming more virulent, and of late, too, it has taken to appear in epidemics. In England an outbreak of 37 cases was reported at Bristol by myself in 1911, and F. E. Batten collected the records of 70 epidemics, affecting 17,500 patients in different countries. Since then outbreaks have been noted in Nottingham, Leicester, Dorset, Devon and Cornwall, Norfolk and Suffolk, and the Edinburgh district, all of them comparatively small, but with a high mortality; whilst in Sweden the last huge outbreak had, up to August, attacked 5,000 persons.

The disease was first of all described by Heine in 1840, the lesions in the anterior horns were reported by Prèvost and Vulpian in 1866, Strumpell in 1885 first pointed out the cerebral cases, while Wickman in 1905 analysed an epidemic and showed the infectiousness of the complaint. As to the general symptoms, there is (1) an *Incubation period* of, probably, three or four days in man. Müller, indeed, would extend it to five or ten days, but the majority of observers favour the shorter limit. In monkeys a fixed period of seven or eight days is found if the virus has been "steadied" by passing it through several animals. (2) Next there is a *Preparalytic period* between the first symptoms and the onset of paralysis, which is important in view of the possibility of treatment to prevent the development of paralysis. The duration of this stage is usually from one to seven days, but in some cases no preparalytic symptoms are perceptible. We may classify these symptoms as (*a*) those which are only prominent in some epidemics, as, for example, the papular rash in one of the New York outbreaks, gastro-intestinal or respiratory troubles, and sore throat; (*b*) the more or less constant ones in all outbreaks, such as pyrexia for some days, often reaching to 103°, with a slight morning fall, irritability, or, on the other hand, drowsiness and stupor, pains in the limbs, especially when passive movements are tried, hyperæsthesia, moderate headache and stiff neck, a remarkable muscular weakness, sometimes twitchings, and finally a curious change in the deep reflexes, which may be increased to-day and decreased to-morrow, or lost in one knee and exaggerated in the other, or lost in both. E. F. Buzzard lays stress on the diagnostic importance of pain, when passive movement is tried, coming on after the great muscular feebleness. Another aid to diagnosis is the state of the spinal fluid. The spinal fluid before paralysis shows:—

1. The absence of all special bacteria, such as those of tuberculosis and cerebro-spinal fever.
2. A moderate increase of pressure.
3. A marked leucocytosis, either polymorphonuclear or lymphocytic.
4. In rare cases an increase of globulin.

Flexner also asserts that when the fluid is opalescent and the Noguchi reaction is positive the case will go on to paralysis. It has also been asserted by Müller and others that the blood shows a leucopenia, but this has not been verified by later research.

(3) *The Third Stage, that of Paralysis*, usually follows directly after these symptoms. Sometimes there is an intermission for a day or more, and sometimes a state of stupor for one or two days; and at times, as we have said, no prodromal symptoms may have been observed. I need hardly point out that a great variation in the types of paralysis is recognised at the present day, and, whereas formerly only spinal cases were included, now many writers, as, for instance, Peabody, Draper, and Dochez, reckon the following:—

1. The cerebral or spastic hemiplegias of Strumpell.
2. The common flaccid or bulbo-spinal paralyses.
3. The abortive or transient ones.

Wickman, the Swedish authority, and others add:

4. An ascending form resembling Landry's paralysis as well as a descending one.
5. An ataxic form due to cerebellar lesions.
6. A meningeal form which is liable to be confused with cerebro-spinal fever.
7. A polyneuritic form resembling peripheral neuritis.
8. A purely bulbar form, separated from group (2) and confined to affections of the cranial nerves and those of respiration and deglutition.

Great discussion has taken place as to the cerebral, or, strictly speaking, cortical paralyses, which are comparatively rare. This type has not been produced in monkeys, and though in epidemics spastic and flaccid paralyses have been seen side by side, or even in the same patient, and though the serum of a spastic case will neutralise an active virus as well as the other does, yet there has rarely been direct proof that the paralysis was due to a cortical lesion. However, definite cases have been found, *e.g.* Harbitz and Scheel reported one with lesions in the right temporal lobe and both gyri (*Monograph, No. 4, Rockfeller Institute*, p. 74).

The abortive cases are highly important, since it is clear that they can transmit the disease. They are true instances of the disease, for (1) they show the ordinary symptoms of the preparalytic stage, and their blood and spinal fluid conform to the tests; (2) some of them show transient paralyses, and others marked muscular weakness; (3) if several monkeys are inoculated with a virus, some of them show all the symptoms, including paralysis, and die; others show all except paralysis, and, perhaps, die also, but they can transmit the disease to others, and when they do recover their serum neutralises a virus so that it no longer reproduces the disease.

The numbers of these abortive cases are large. Müller would go so far as to say that they outnumber the cases of paralysis. Wickman claims that in his epidemic they were from a quarter to a half of the whole number. Indeed, until the recognition of the disease in the early stages is made easy and certain, many cases will escape us.

The locality of the paralysis depends on the lesions in the grey matter. These lesions are generally produced in the spinal cord by the exudate into the perivascular lymph spaces of the blood-vessels. Now these vessels are most abundant in the anterior horns of the cervical and lumbar enlargements. Hence the commonest paralysis is that of a group of muscles innervated from segments in one of those enlargements. The legs suffer more often than the arms, and the proximal segments than the distal ones, and they recover less often. Thus the deltoid group is very often attacked and is apt not to recover.

The spinal fluid after paralysis has occurred is:

1. Clear, colourless and watery.
2. No bacteria are present in it.
3. There is an increased amount of globulin present.
4. Some increase of pressure is noticeable.
5. Fehling's fluid is always reduced.
6. An increased number of cells is present, almost always of the mononuclear type.

These characters are generally sufficient to exclude cerebro-spinal and other forms of meningitis. The blood counts in this stage have been much disputed. Müller, Gay, and Lucas insist that there is a leucopenia with lymphocytosis. Peabody and his colleagues examined fifty-nine cases and found almost always a marked leucocytosis with an increase of polymorphonuclears and a decrease of lymphocytes.

Pathology.—The changes outside the nervous system show some likeness to those in typhoid. The parenchymatous organs are marked by "cloudy swelling." In the liver one sees numerous small groups of cells which have suffered a hyaline necrosis, while a considerable proliferation has gone on around them and a general tendency towards cirrhosis. In the lymphoid tissues we find marked overgrowth both of Peyer's patches, of the mesenteric lymph nodes, and to a less extent of the axillary and other glands, of the tonsils, spleen, and thymus. As to the spinal cord, there is œdema, cellular infiltration, and hæmorrhages. Collections of small mononuclear cells are seen in the lymph spaces, sometimes forming an obstructive collar around the vessels; hæmorrhages also take place from these vessels and cause necrosis of the nerve cells in the anterior cornua. Petterman finds in some cases necrosis of the cells without previous vascular changes. Similar but slighter changes are seen in the posterior root ganglia, in the white matter, and also in the cerebrum, medulla, and pons.

We now come to the question, What is the *Infecting Agent?* We find that it belongs to a special class, that it is ultra-microscopic like the organisms of yellow fever, rabies, and pleuro-pneumonia and a dozen other diseases, yet much exact knowledge of it has been gained since Landsteiner reproduced the disease in 1909 in monkeys. It is highly resistant, being uninjured by freezing for weeks, or drying, or by .5 per cent. carbolic acid, or by prolonged contact with glycerine; but it is killed by heating to 45° or 50° C. for half an hour, by menthol, pot. permanganate, $H_2O_2$ in 2 per cent. strength, or hydrarg. perchlor.

Its entrance into the human body is through the nasal mucous membrane, as Flexner and Lewis proved. It is never found in the human blood, spinal fluid, spleen, or liver, but almost exclusively in the central nervous system, the tonsils, mesenteric glands, and nasal mucosa. It leaves the body partly by the nose, for it is found in the secretions, and indeed organisms injected into the spinal canal easily pass down the lymph channels which accompany the olfactory nerve. The Swedes have also shown the organism in the lining of the small intestine after death, and in washings from the colon during life, so that clearly the dejecta may be infectious. So far, however, the virus has not been found in the urine or fæces, and healthy monkeys cannot be infected by feeding them on infected food, unless their bowels are first paralysed by opium. Flexner has recently shown that the virus survives the action of the stomach for two hours (*J. A. Med. Assoc.*, July 27).

One attack in the monkey or man usually gives life-long immunity. Wickman found that the serum of patients who had recovered three years before destroyed the virus, and in this way he proved the unity of the sporadic and epidemic disease. Immunity to many times a lethal dose may be gained by repeated injection of a weakened virus as in rabies. The production of a serum or vaccine for treatment has so far been a failure. Experimental poliomyelitis is only successful as a rule in monkeys; horses and sheep never develop the disease though they produce immune bodies in their serum.

Rabbits occasionally reproduce the disease, but Marks has found that the virus can produce in them a disease with no symptoms of myelitis, which can be passed through several rabbits and back again to monkeys, when the old type of disease reappears. Thus a "reservoir" so called may possibly exist in some domestic animals which can transmit the disease without showing the symptoms.

Indeed it is clear that human carriers do exist, either immune persons, recovered or abortive cases. Thus Petterson and his assistants found the organism in the nose and throat of healthy persons who had been in close contact with patients, and also in the throats of abortive and ambulant cases. They show too that nine of their convalescents were infectious for from four to seven months. Osgood and Lucas found the virus in another case five months after the disease, though usually in monkeys it dies out in three weeks, and indeed Flexner, Peabody, and others lay down that four weeks is a proper limit for isolation. In short, the majority of people probably cease to be infectious in four weeks, but some may remain life-long carriers.

When death occurs in the early stages, it is practically always from respiratory failure, that is, from a bulbar paralysis, or paralysis of phrenics or intercostals, and not from toxæmia. A paralysis affecting the deltoid group, or a rapidly ascending one, may quickly become dangerous, especially if the patient has been very prostrate from the beginning. Death most commonly happens on the fourth day of paralysis, but the danger is not over till the eighth. The rate of mortality varies from 10 to 25 per cent., or 11.9 for children and 27.6 for adults, as Wickman estimated. Of course, if the abortive cases are really very frequent then these rates are too high.

There is no proof that the various forms of paralysis in domestic animals are due to this disease. The domestic fowl often suffers from paralyses, but these are due to a peripheral neuritis. The *house fly* can carry the virus for forty-eight hours, but outbreaks do occur in the winter when he is absent. The mosquito, the louse, and the flea have so far failed to take the infection, but the bug has succeeded, and a filtrate from the infected animals conveyed the disease to monkeys. Spiller mentions a man who was bitten on the foot by an insect (? a bug) and developed paralysis (*Penns. Med. Jour.*, 1911). There is no doubt that infection is important, though its action appears most capricious from our ignorance of its laws. Probably there is a very high resistance in most individuals, and the organism can only get a foothold in the few less protected. In some epidemics nearly half the houses show more than one case each. In Trastena it was thirteen out of nineteen. In Devonshire there were instances of three, four, and six cases in one house, and there are records of persons attacked days after a single contact with an infectious person. *Dust*, too, has been found to convey the infection. Neustader took the dust from the room of a patient, filtered a solution of it through porcelain to remove all bacteria, injected it into monkeys and produced paralysis and the usual lesions. Wickman gives instances where the disease arose in the same house for two or three successive years.

Some confusion has existed between cerebro-spinal fever and poliomyelitis. This is partly due to the old idea that the latter is a painless paralysis and not a fever. Again, outbreaks of both disease have occurred in the same place together, as in Leicester in 1910. The initial symptoms and the mildest cases of each disease are not unlike, but on the other hand we have the following differences adapted from Dr. Reece's table:—

| *Cerebro-spinal.* | *Poliomyelitis.* |
|---|---|
| 1. Is at its maximum in April. | 1. Is at its maximum in August. |
| 2. Spinal fluid is very turbid with much albumen, no sugar, but many diplococci and polymorphonuclears. | 2. Is clear, colourless, reduces Fehling, shows no bacteria, and only mononuclear cells after the first few days. |
| 3. The age incidence is later. | 3. Is earlier. |
| 4. Meningeal symptoms are marked and very common. | 4. Are rarely seen. |
| 5. Paralysis is not common. | 5. Permanent paralysis is seen in from 16 to 56 per cent. |
| 6. Fever is prolonged and irregular. | 6. Fever only lasts from one to seven days. |
| 7. Coma frequent. | 7. Drowsiness may be profound, but patient is sensible when roused. |
| 8. Herpes is frequent and often bilateral. | 8. Not seen on patients, but possibly common in abortive cases or healthy persons during an epidemic. |
| 9. The pia mater is infiltrated by polymorphic cells. | 9. The pia is infiltrated by lymphocytes. |
| 10. Blindness and deafness are common sequelæ. | 10. Neither occurs as a sequela. |

Treatment.—The aim, of course, must be to check the spread of the disease and to neutralise the poison before the stage of paralysis. With our present knowledge much can be done to stop the infection, but we have not obtained a vaccine. Flexner and Clark showed that if the virus was injected into monkeys whose spinal fluid contained urotropin, and if this drug was given continuously afterwards, the onset of the disease was postponed in some, and in others no paralysis occurred. Hence it has been given in the early stages, and at Stockholm it is said that good results have been obtained. It is well-known that when given by the mouth some is excreted into the spinal fluid and the gall-bladder. The difficulty is to give sufficiently large doses, but S. J. Crowe of Johns Hopkins finds that very large quantities, such as 40 to 160 grains per diem, can be easily taken, if divided into many doses and well diluted, such as one grain in every ounce of liquid swallowed, or if administered as a rectal injection. At that hospital it has been given as a routine method since 1908 in poliomyelitis, ear infections, and compound fracture of the skull. It seems important to avoid exertion during the acute stage as this appears to have brought on paralysis in some abortive cases. Disinfecting nasal sprays are desirable for all persons in contact with patients, and indeed for patients themselves. When the pain is severe lumbar puncture may give relief and so do hot baths.

References.

Batten, F. E, Royal Society of Medicine, 1911. Batten, F. E., *Lancet*, 1912. i., 413. Clark, *American Journal of Medical Science*, 1912. i., 721. Trethowan, *Lancet*, 1912, ii., 938. S. J. Crowe, *Johns Hopkins Bulletin*, 1912. Sophian, *Archives of Pediatrics*, New York, 1912, p. 165. Munro, *Medical Press and Circular*, 1912, i., p. 82. Hernaman Johnson, *British Journal of Children's Diseases*, 1911, p. 539. Lewis, *Pennsylvania Medical Journal*, 1911-12, pp. 173-196. Reece, Farrar, Mervyn Gordon, and Macewen, *Local Government Board Report*, No. 61. S. Flexner, *Journal of American Medical Association*, 1912, October 12. J. G. Forbes, *Lancet*, 1911, ii., 1400. E. F. Buzzard, *Lancet*, 1912, i., p. 23. Peabody, Draper, and Dochez, *Monographs of the Rockefeller Institute*, No. 4, 1912, June. S. Flexner, Notes 1 to 13 from the *Rockefeller Institute*. F. A. Williams, *Pennsylvania Medical Journal*, 1911, December, p. 196.

